# A Novel Case of Functional Gastric Neuroendocrine Carcinoma Occurred after Endoscopic Submucosal Dissection

**DOI:** 10.1155/2013/148761

**Published:** 2013-11-06

**Authors:** Yoshiaki Shibata, Yuji Ito, Hiroko Fujita, Yume Okada, Toshihiko Nagai, Hirohito Sano, Kumiko Ito

**Affiliations:** ^1^Division of Gastroenterology, Tama-Hokubu Medical Center, Tokyo Metropolitan Health and Medical Treatment Corporation, 1-7-1 Aobacho, Higashimurayama-shi, Tokyo 189-0002, Japan; ^2^Division of Pathology, Tama-Hokubu Medical Center, Tokyo Metropolitan Health and Medical Treatment Corporation, 1-7-1 Aobacho, Higashimurayama-shi, Tokyo 189-0002, Japan; ^3^Division of Endocrinology and Metabolism, Tama-Hokubu Medical Center, Tokyo Metropolitan Health and Medical Treatment Corporation, 1-7-1 Aobacho, Higashimurayama-shi, Tokyo 189-0002, Japan

## Abstract

In Japan, endoscopic submucosal dissection (ESD) is becoming a standard treatment for intramucosal differentiated gastric cancer. Although ESD is associated with a high cure rate for patients with early gastric cancer, tumors may recur, albeit rarely. We performed ESD on an 80-year-old man with a small depressed type of gastric cancer of the posterior wall of the cardia, found to be locally invasive on histology. Thirty months later, local recurrence and multiple liver metastases were detected, accompanied by frequent severe hypoglycemia. Despite chemotherapy, the patient died 6 months after relapse. On autopsy, the recurrent gastric lesion and liver metastases were examined immunohistochemically. Several characteristic tumor cells were positive for chromogranin A, cluster of differentiation (CD) 56, Ki-67, and insulin-like growth factor (IGF)-II. Western blot analysis of the patient's serum obtained during a hypoglycemic attack showed the high molecular weight form of IGF-II or “big” IGF-II. The patient was diagnosed with non-islet cell tumor hypoglycemia (NICTH), with “big” IGF-II being produced by the gastric neuroendocrine carcinoma. This is the novel case of a functional gastric neuroendocrine carcinoma that occurred after ESD and induced a hypoglycemic attack associated with NICTH.

## 1. Introduction

Tumor-associated hypoglycemia was recently shown to be caused by high molecular weight insulin-like growth factor (IGF)-II or “big” IGF-II [1–5], a condition referred to as non-islet cell tumor hypoglycemia (NICTH). This condition has been reported in patients with mesenchymal tumors but rarely in patients with gastric cancer [4, 5]. Gastric neuroendocrine carcinomas are rare, but their development and progression process or association with NICTH is not clear. We report a very rare case of a functional gastric neuroendocrine carcinoma producing “big” IGF-II and liver metastases as well as NICTH, after endoscopic submucosal dissection (ESD). Through the clinical course and autopsy findings of this patient, we approached the nature of the disease.

## 2. Case Presentation

**Figure 1:**
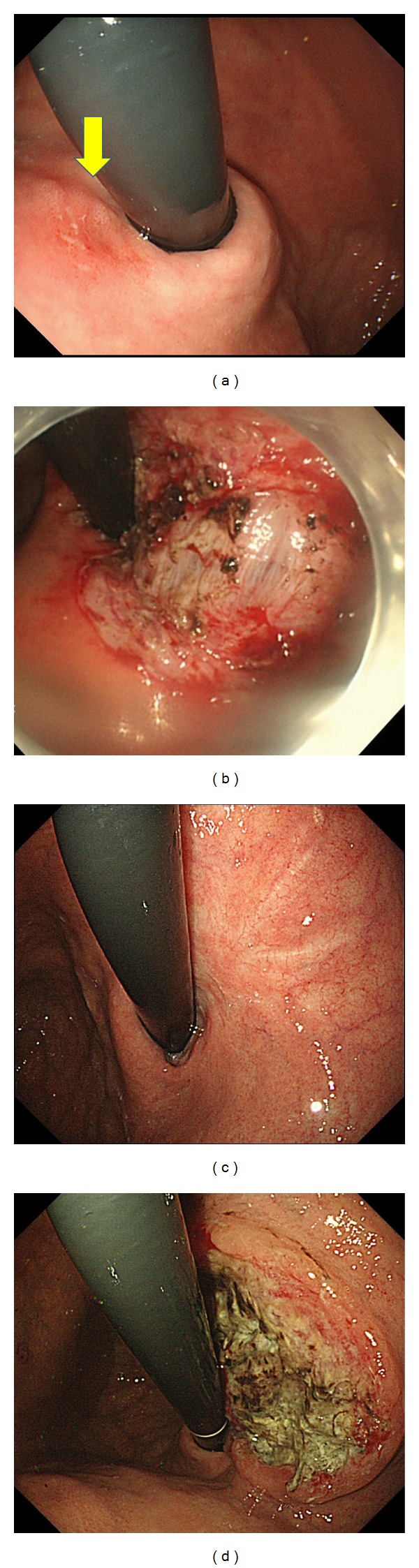
(a) Endoscopic findings of the first EGD revealed the small depressed type of early gastric cancer of the posterior wall of the cardia (arrow). (b) Endoscopic findings after submucosal dissection. En block resection was obtained by ESD method. (c) Endoscopic image 24 months after ESD. There was no evidence of recurrence. (d) Endoscopic image 30 months after ESD. Local recurrence of the type 2 advanced gastric cancer was found.

**Figure 2:**
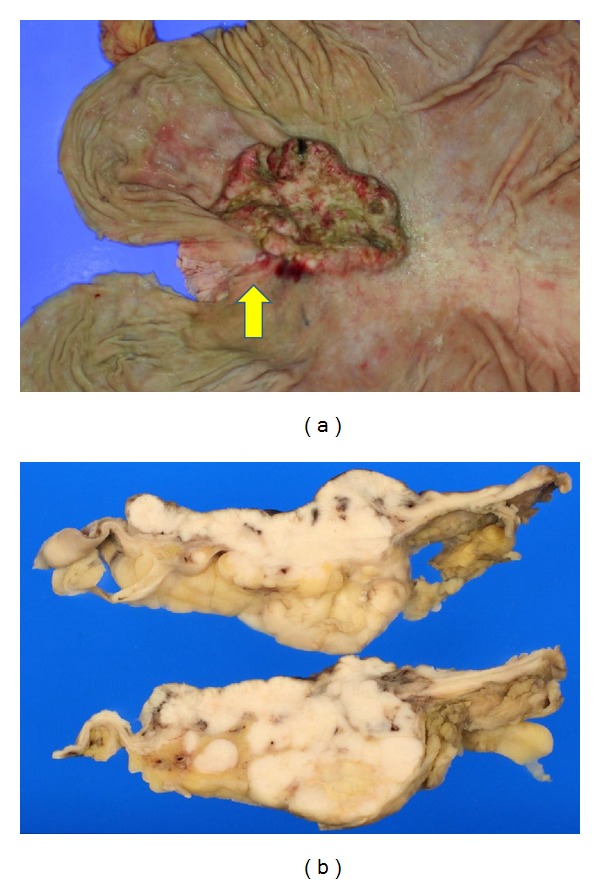
(a) Gross feature of recurred gastric lesion. The type 2 tumor, measuring 6 × 6.5 × 2.5 cm, was located in contact with the scar (arrow) after ESD. (b) The cut surface of gastric tumor revealed solid whitish tumor with infiltration of the serosa.

**Figure 3:**
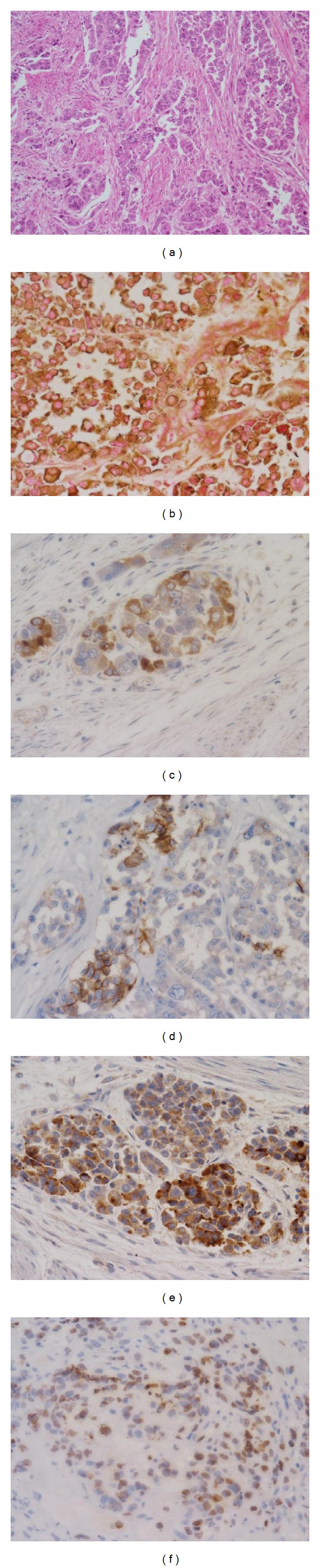
Histological features of the tumor cells. Grimelius and various immunohistochemical staining techniques were positive. (a) HE. (b) Grimelius. (c) Chromogranin A. (d) CD56. (e) IGF-II. (f) Ki-67.

**Figure 4:**
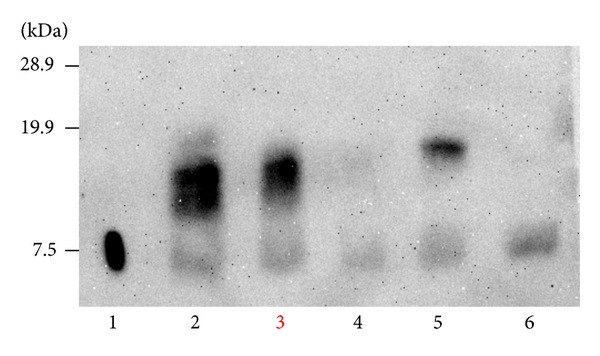
Western immunoblot analysis of serum IGF-II. Patient (Lane 3) and NITCH (Lanes 2 and 5) revealed a high molecular weight form of IGF-II compared with recombinant IGF-II (7.5 kDa; Lane 1) or normal (Lane 6).

An 80-year-old man underwent ESD in our hospital for a small depressed type of early gastric cancer of the posterior wall of the cardia (Figures 1(a) and 1(b)). The resected tumor was 12 mm in diameter, and results of histological examination showed that it consisted mainly of moderately differentiated adenocarcinoma, with partly mucinous carcinoma. Although microinfiltration into the submucosa was observed, tumor invasion was absent in both horizontal and vertical margins, and there was no evidence of lymphatic vascular invasion. The patient refused additional gastrectomy but underwent endoscopy and computed tomography (CT) examinations every 6 months. There was no evidence of local recurrence or distant metastases for 24 months after ESD, but esophagogastroduodenoscopy at 30 months showed a type 2 advanced gastric cancer approximately 5 cm in diameter on the ESD scar (Figures 1(c) and 1(d)). Biopsies showed that this tumor was a poorly differentiated adenocarcinoma, and CT showed multiple liver and para-aortic lymph node metastases. Although he was started on chemotherapy with S1 (100 mg on days 1–21) and cisplatin (60 mg on day 8), liver metastases increased. Around the same time, he started getting cold sweats early in the morning. Measurement of blood glucose concentration showed marked hypoglycemia, less than 20 mg/dL (reference range, 70–109 mg/dL). After admission, he received a continuous infusion of glucose, but his hypoglycemia did not improve. He was started on second-line paclitaxel chemotherapy (80 mg on days 1, 8, and 15 every 4 weeks). CT showed a partial response of the liver metastases and a gradual reduction in hypoglycemia after one round of chemotherapy, but regrowth of these metastases and hypoglycemia occurred after two rounds, and he died 6 months after cancer recurrence from hypoglycemia. An autopsy showed a type 2 tumor, measuring 6 × 6.5 × 2.5 cm, in contact with the ESD scar (Figures 2(a) and 2(b)), and multiple liver metastases. Histological examination showed serosal invasion of diffusely infiltrating large tumor cells, with marked nuclear pleomorphism, forming nests or sheet patterns with central necrosis (Figure 3(a)). The cells were positive on Grimelius silver staining, a method of detecting neuroendocrine granules (Figure 3(b)). Immunohistochemical analysis of the tumor cells showed that they were positive for chromogranin A, CD56, and IGF-II (Figures 3(c)–3(e)). The Ki-67 labeling index, an index to estimate cell proliferation, was 43.6% (Figure 3(f)), higher than the cutoff (>20%) for neuroendocrine carcinoma defined by the WHO [6]. Western blot analysis of patient serum showed an IGF-II band of 19.8 kD larger than the 7.5 kD band observed in healthy individuals (Figure 4).

## 3. Discussion

NICTH is a rare disease, characterized by severe hypoglycemia associated with tumors, except for insulinomas [1–5]. For high molecular weight, partially processed precursors of IGF-II have structural and functional properties similar to those of insulin [1–5]. “Big” IGF-II produced by tumors can bind to insulin receptors, resulting in hypoglycemia [3]. Our patient did not have an insulinoma, because his serum insulin concentration was reduced during hypoglycemic attacks. Measurements of cortisol and growth hormone (GH) concentrations excluded adrenal and pituitary insufficiencies. 

Western blot analysis showed the presence of “big” IGF-II, rarely detected in healthy individuals, confirming a diagnosis of NICTH. His primary gastric lesion and liver metastases were immunohistochemically positive for IGF-II and neuroendocrine markers. According to the revised WHO classification in 2010, pancreatic and gastrointestinal tumors with endocrine properties are collectively called neuroendocrine neoplasms and can be divided into 3 major categories, Grades 1 and 2 neuroendocrine tumors (NETs) and neuroendocrine carcinomas (NECs), depending on their degree of proliferation and malignant potential, measured using the Ki-67 labeling index [6], with NECs having a Ki-67 labeling index >20%. NECs can be further subdivided into small cell and large cell neuroendocrine carcinomas (LCNECs). Our patient was diagnosed with LCNEC, because of the atypical features of the large tumor cells and the high Ki-67 labeling index [6, 7]. LCNEC is a rare histological type, accounting for approximately 1.5% [7] of all gastric cancers. Because the malignant grade of LCNEC is very high, as shown by early vascular invasion and liver metastases, prognosis is very poor [7]. Overproduction of “big” IGF-II not only causes hypoglycemia but also is associated with tumor growth [1]. In our patient, hypoglycemia was not observed at the time of cancer recurrence or during second-line chemotherapy but was associated with the increase in liver metastases. This fact shows that hypoglycemia attack induced by “big” IGF-II production has been related with tumor growth. Most patients who experience local recurrence after ESD for early gastric cancer do so within one year [8], with local recurrence after 2 years being rare. In fact, we tried to immunostain the specimens resected endoscopically, but endocrine markers were not stained. It is well known that cancer can exhibit various histological types through a process of the growth. Although the tissue type of the primary and secondary tumors differed in our patient, the secondary tumor was located at the ESD scar, and tumor invasion had advanced in the vertical direction, indicative of local recurrence. After ESD, a very small number of cancer cells may have remained in the stomach wall, hidden, because of the effects of heat denaturation. These remnant cancer cells may have acquired the ability to produce “big” IGF-II during the process of differentiation and development into endocrine cell carcinoma. Ida et al. reported gastric neuroendocrine carcinoma presenting with NICTH, but large molecular weight IGF-II could not be proved [9]. Therefore, this is the first report that demonstrated “big” IGF-II production in gastric neuroendocrine carcinoma associated with NICTH. Because the cell morphology of some gastric NICTHs is similar to that of LCNEC, neuroendocrine markers should be assayed when NICTH is suspected. Some of the gastric neuroendocrine carcinomas may be proposed as a new concept of disease: functional neuroendocrine carcinoma associated with “big” IGF-II production induced hypoglycemia. Our findings provide insight into the processes of gastric carcinogenesis and progression and suggest that the use of ESD in patients with gastric cancer be tailored according to histological type and depth of cancer invasion.
